# Promising Nanotechnology Approaches in Treatment of Autoimmune Diseases of Central Nervous System

**DOI:** 10.3390/brainsci10060338

**Published:** 2020-06-02

**Authors:** Maria Chountoulesi, Costas Demetzos

**Affiliations:** Section of Pharmaceutical Technology, Department of Pharmacy, School of Health Sciences, National and Kapodistrian University of Athens, Panepistimioupolis Zografou, 15771 Athens, Greece; mchountoules@pharm.uoa.gr

**Keywords:** multiple sclerosis, nanotechnology, drug delivery nanosystems, lipids, polymers, vaccines, nanoparticles, antigen-specific immunotherapy, experimental autoimmune encephalomyelitis, neurodegeneration

## Abstract

Multiple sclerosis (MS) is a chronic, autoimmune, neurodegenerative disease of the central nervous system (CNS) that yields to neuronal axon damage, demyelization, and paralysis. Although several drugs were designed for the treatment of MS, with some of them being approved in the last few decades, the complete remission and the treatment of progressive forms still remain a matter of debate and a medical challenge. Nanotechnology provides a variety of promising therapeutic tools that can be applied for the treatment of MS, overcoming the barriers and the limitations of the already existing immunosuppressive and biological therapies. In the present review, we explore literature case studies on the development of drug delivery nanosystems for the targeted delivery of MS drugs in the pathological tissues of the CNS, providing high bioavailability and enhanced therapeutic efficiency, as well as nanosystems for the delivery of agents to facilitate efficient remyelination. Moreover, we present examples of tolerance-inducing nanocarriers, being used as promising vaccines for antigen-specific immunotherapy of MS. We emphasize on liposomes, as well as lipid- and polymer-based nanoparticles. Finally, we highlight the future perspectives given by the nanotechnology field toward the improvement of the current treatment of MS and its animal model, experimental autoimmune encephalomyelitis (EAE).

## 1. Introduction

Multiple sclerosis (MS) is a chronic, autoimmune, demyelinating disease of the central nervous system (CNS), accompanied by a relapsing/remitting (RR) or a progressive course that is followed by axon damage and paralysis, including symptoms of muscle weakness, weak reflexes, muscle spasm, difficulty in movement, miscoordination, unbalance, vertigo, fatigue, and pain. Other symptoms that are usually referred are optic nerve dysfunction, loss of vision, diplopia, pyramidal tract dysfunction, ataxia, tremor, bladder and bowel dysfunction, sexual dysfunction, depression, anxiety, swallowing dysfunction, memory loss, sleep disturbance, and obstructive sleep apnea [1,2,3,4,5]. Unfortunately, the exact etiology of MS remains unknown, while many different risk factors were referred, characterizing MS as a heterogeneous, multifactorial disease. The occurrence is 2–3 times higher in females than males. MS is the most common neurologically disabling disease in young adults, while older people and children can also acquire MS [4,6]. Our understanding of the immune processes that contributes to MS led to the approval or clinical development of some disease-modifying therapies (DMTs) that are effective in relapsing forms of MS. However, few treatments are effective for the progressive forms of the disease [7,8].

Nanotechnology provides a variety of promising therapeutic tools that can be applied for the treatment of CNS-related disorders, such as MS, overcoming the barriers and the restrictions of the already existing conventional therapies. Extensive research is being carried out for the development of drug delivery nanosystems for the targeted delivery of MS drugs in the pathological tissues of CNS, providing high bioavailability and enhanced therapeutic efficiency. In addition, remyelination is an attractive, innovative strategy toward MS therapy [9], where nanoparticles can also contribute, via the targeted delivery of remyelinating agents to specific cells, leading to the improvement of their therapeutic performance. Moreover, tolerance-inducing vaccines, based on tolerance-inducing nanocarriers for antigen-specific immunotherapies, are considered to be another promising strategy toward the treatment of MS [10,11].

In the present review study, literature examples of the aforementioned nanocarriers that were designed for MS treatment are presented, highlighting the future perspectives given by the nanotechnology field toward the improvement of the current treatment of MS. We focus on liposomes, as well as lipid- and polymer- based nanocarriers.

## 2. Multiple Sclerosis (MS)

MS is an autoimmune, chronic, neurodegenerative disorder, targeting the myelin sheaths (a protective layer surrounding the nerve fibers) of the CNS. The caused damage of myelin sheaths provokes nerve demyelination, followed by axon damage and, thus, interruption of signal transmission to and from the CNS. As with many other neurodegenerative diseases, the real and exact origin of MS is still unidentified, although the literature describes many different potential triggering factors that may stimulate the autoimmune responses, which harm the brain tissues and spinal cord. More particularly, genetic predisposition and environmental factors, as well as microbial and viral infections, smoking, toxins, low concentrations of vitamin D, and circadian rhythm disruption, can contribute to the onset of this disorder [12,13,14,15,16]. Regarding genetic predisposition, the major histocompatibility complex (MHC) class II phenotype, the human leukocyte antigen (HLA)-DR2, and HLA-DR4 are reported as the most commonly affected, while the incidence of MS is also increased 10-fold in monozygotic twins, as compared to siblings of patients with MS [17,18].

MS is categorized into three distinct types, primarily based on its clinical course, which are characterized by increasing severity. Relapsing/remitting MS (RRMS) is the most common form, which involves relapses followed by silent remission with any MS symptoms. RRMS generally switches to a chronic progressive course several years after onset, while a minor number of patients display primary chronic progressive course without the RR phase. Subsequently, we have secondary progressive MS (SPMS), which develops over time following diagnosis of RRMS, and primary progressive MS (PPMS), noted as gradual continuous neurologic deterioration, which provides continuous disease progression [19,20,21].

The symptoms may vary person-to-person and produce temporary, long-lasting, or even permanent losses due to the disrupted signal transmission, including mood swings, memory-related issues, tingling, fatigue, numbness, partial or complete blindness, pain, and partial or even whole-body paralysis, depending upon the severity of disease. The disrupted signal transmission is responsible for various complex and erratic indications. MS broadly presents three stages, starting from a pre-clinical stage, followed by a relapsing/remitting stage and a progressive clinical stage, in which, after typically 10–20 years, neurologic dysfunction progressively worsens, eventually leading to impaired mobility, cognition, and a progressive loss of nerve functions [22,23,24,25].

### 2.1. Immunopathophysiology of MS

**Figure 1:**
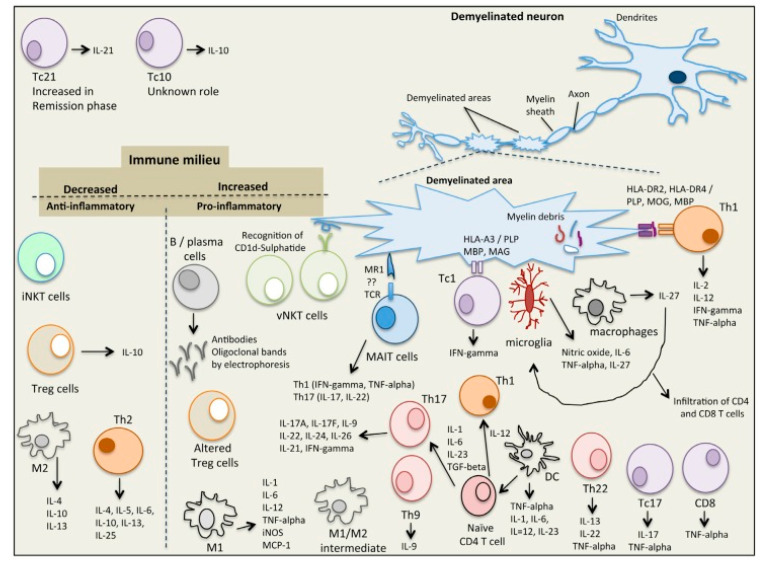
The immunological complexity of the immune/cytokine network in multiple sclerosis (MS). Adapted from Dargahi et al. [25].

Myelin sheath damage and inflammation contribute to the formation of lesions [26]. MS lesions appear in the white matter inside the visual neuron, brain stem, and spinal cord [26]. In MS, the immune system starts to recognize myelin components as foreign, leading to its destruction [12]. Plaque formation and disease symptoms are widely accepted as the result of immune cell infiltration, with the release of cytokines and inflammatory mediators, leading to inflammation, myelin destruction, oligodendrocyte loss, and loss of neuronal function and eventual axonal degeneration. On the other hand, demyelination further increases the activation of inflammatory processes, causing damage of the blood–brain barrier (BBB), stimulation of oxidative stress pathways, and macrophage activation [27]. Microglial cells upregulate MHC class I and II molecules, as well as cell surface co-stimulatory molecules, while they also secrete cytokines and chemokines. Several types of immune infiltrates can be found in the white matter lesions, where myelin is damaged, including monocytes, B cells, T cells (for example, TH cells and myelin-reactive auto-T cells), and dendritic cells [25,28,29,30,31,32]. Substantial fractions of cluster of differentiation 4 (CD4)+ and CD8+ T cells being isolated from MS lesions and cerebrospinal fluid (CSF) indicate that antigen-specific T-cell responses contribute to the disease process [33]. It seems that T helper 1 (TH1) and TH17 cells are the main pathogenic populations in the immunopathogenesis of MS, along with regulatory T cells (Treg) and natural killer T (NKT) cells [34,35,36,37]. The pro-inflammatory state of macrophages is correlated with MS because macrophages interact with T and B cells, instructing demyelination, axonal loss, and degeneration, and they are presented as of the most predominant cell type in patient lesions [38,39]. In Figure 1, there is a detailed presentation of the cells that are involved in the immune process of MS [25].

Traditionally, the etiology of MS was based on an “outside-in” autoimmune hypothesis, whereby dysregulated auto-reactive T cells in the periphery cross into the CNS parenchyma and, together with macrophages and B cells, proceed to attack myelin. Contrariwise, the “inside-out” hypothesis argues that MS is a primary degenerative disease, where the initial malfunction occurs within the CNS and is accompanied by varying degrees of inflammation, as a secondary response, leading to the release of various antigenic cell components. According to the “inside-out” hypothesis, the primary degeneration is present from the start (probably years before the first overt clinical symptoms) and continues throughout the entire course of the disease. Whether early neurodegeneration drives autoimmune injury, or whether ongoing inflammation reaches a threshold to trigger neurodegeneration is still unclear, while another question is whether neurodegeneration is independent or not of chronic inflammation. The lack of understanding with regard to mechanisms of progression phase, when the most irreversible disability takes place, is responsible for the extremely limited treatment options that are currently available to patients with progressive MS. Until now, many different treatment approaches for progressive MS were proposed including mitochondrion-protective strategies, anti-inflammatory strategies, strategies targeting microglia and astrocytes, inhibitors of microglial activity, remyelination therapies, and strategies targeting lymphocytes [20,23,40].

### 2.2. Current Therapeutics of MS

Until now, the Food and Drug Administration (FDA) approved over a dozen therapeutic agents to reduce the number of attacks and delay MS progression in terms of available DMTs. Disease-modifying agents are commonly shown to reduce the rate of relapses, reduce magnetic resonance imaging (MRI) lesions, and stabilize or delay MS disability. However, MS remains incurable. Interferon beta (IFN-β) and glatiramer acetate (GA), being the first two introduced drugs, are able to alter T-cell responses, are injectable, and still remain the “first line” therapies for MS, owing to their relative safety and proven efficacy. Oral DMTs available for RRMS include immunosuppressives, which inhibit lymphocyte trafficking, namely, fingolimod and the teriflunomide (TFM) that inhibit activated T and B cells, as well as the immunomodulatory/immunosuppressive dimethyl fumarate (DMF) that alters T-cell responses. The above are characterized as long-term treatments. In addition, a number of humanized monoclonal antibody-based therapeutics for RRMS were developed, including natalizumab, alemtuzumab, and ocrelizumab. Unfortunately, their disadvantage is the high risk of side effects, including progressive multifocal leukoencephalopathy (PML) and the development of secondary autoimmune diseases [4,12,25,26,39,41,42,43,44,45].

### 2.3. Experimental Autoimmune Encephalomyelitis (EAE) as an Animal Model for MS

Experimental autoimmune encephalomyelitis (EAE) is a widely used animal model of MS because it shares several features with the human disease, including neurological dysfunction and perivascular inflammation in the CNS. It is considered to be the most frequently used model to study the demyelinating and immune pathology of MS. EAE can be induced in several mammalian species by directly immunizing animals with CNS homogenate or myelin proteins, such as myelin oligodendrocyte glycoprotein (MOG), myelin basic protein (MBP), and proteolipid protein (PLP), or using small peptides derived from these proteins; alternatively, the transfer of isolated activated CD4+ T cells or less commonly CD8+ T cells, may be introduced to a naïve animal. The fact that demyelination and lesion formation occur predominantly in the spinal cord rather than the CNS is a disadvantage indicating that EAE does not fully recapitulate human MS pathology [46,47,48,49,50]. However, there is no other model representing better the pathophysiology of MS. EAE extensively contributed toward the knowledge and understanding of the pathophysiology of the MS, as well as contributed to the discovery and the understanding of the mechanism of action of some of the current approved therapies [39,51,52,53].

## 3. Nanotechnology and MS

Drug delivery nanosystems are considered as innovative technological platforms that are able to transport bioactive molecules to target tissues, modifying their solubility and improving their bioavailability, by altering their pharmacokinetic profile [54]. The existence of physiological barriers, such as the BBB and the blood–cerebrospinal fluid barrier (BCSFB), limits the access of several therapeutic agents to the CNS and downgrades their therapeutic activity. In this regard, nanotechnology is considered to be a promising strategy to improve drug targeting to the brain, as well as increase bioavailability. There is great research attention toward the employment of nanotechnology, as well as the development of new therapies and improvement of the therapeutic efficacy for MS [55]. Taking advantage of their size, nanoparticles are easily internalized by the cells, being suitable carriers for drugs, immunomodulatory molecules, or antigens. Nanosystems are able to improve the drug solubility, provide targeted delivery, diminish potential side effects from high doses, and establish controlled drug release. Moreover, nanoparticles can be administrated through various routes apart from systemic administration, such as intranasally, for example, in cases where nose-to-brain delivery is desired.

In the following paragraphs, different literature cases on nanoparticles and MS are examined. There are referred nanoparticles that are used as drug delivery systems, in order to improve the pharmacokinetics and bioavailability, while enhancing the therapeutic efficacy when compared to the free administered drugs; furthermore, nanoparticles can be used as vectors for antigen-specific immunomodulation. Through antigen-specific immunomodulation, the immune system is repeatedly exposed to a specific antigen, which results in immunomodulation from the disease to the tolerance state. Antigen-loaded nanoparticles provide several advantages for antigen-specific immunomodulation, such as sustained antigen release, the co-delivery of antigens and adjuvants, the formation of antigen depots at the injection site, the effective presentation of B-cell epitopes, and an increase in the uptake and stimulation of cell-mediated immune responses against acellular antigens. These nanoparticles are coupled with specific antigens related to the autoimmune response in MS and their epitopes. These nanoparticles are able to regulate T-cell function, as well as induce Treg cell and dendritic cell differentiation, restoring immunological tolerance [56]. More details are analytically described throughout the paragraphs below.

Another subject of great scientific interest regarding MS, which is also correlated with the development of new therapeutics, is remyelination. While some patients suffer from progressive neurological deficits, other patients occasionally display improved neurological function. The mechanism via which some patients experience neurological improvement remains unclear. However, increasing evidence shows that remyelination occurs in the MS/EAE [57,58,59,60]. In addition, recent studies showed that activated neural stem/progenitor cells around region sites contribute to remyelination [61,62]. Promoting remyelination, therefore, provides an additional line of defense against the axonal damage that follows the loss of myelin. Several potential drugs that target CNS inflammation and the different aspects and stages of remyelination (for example, by inducing oligodendrocyte precursor cell (OPC) differentiation) are being identified [9,63]. Nanoparticles themselves can also act as remyelinating agents. Most recently, Robinson et al. [64] reported a new nanocatalytic therapeutic candidate for remyelination, consisting of a suspension of clean-surfaced, faceted nanocrystals of gold, which was found to demonstrate robust remyelinating activity in response to demyelinating agents, in both chronic cuprizone and acute lysolecithin rodent animal models. More analytically, the administrated nanoparticles induced differentiation of OPCs and enhanced activities of neurons and oligodendrocytes through enhancement of bioenergetic processes of key indicators of aerobic glycolysis. According to the in vivo results, the oral delivery of gold nanocrystals improved the motor functions of cuprizone-treated mice in both open-field and kinematic gait studies. Additional in vitro data indicated an upregulation of myelin synthesis-related genes, collectively resulting in functional myelin generation.

However, there is still no available treatment to regenerate myelin, and several strategies are being scrutinized, while there are difficulties in translating these potential drug targets into practical therapies for patients, as these must cross the BBB to reach OPCs in the CNS, and ideally should be delivered directly to OPCs, to avoid off-target effects. Once again, nanotechnology may solve these problems, with nanoparticles crossing the BBB and facilitating targeting to specific cells, as highlighted in the below-described literature examples.

### 3.1. Lipid-Based Nanosystems

**Figure 2:**
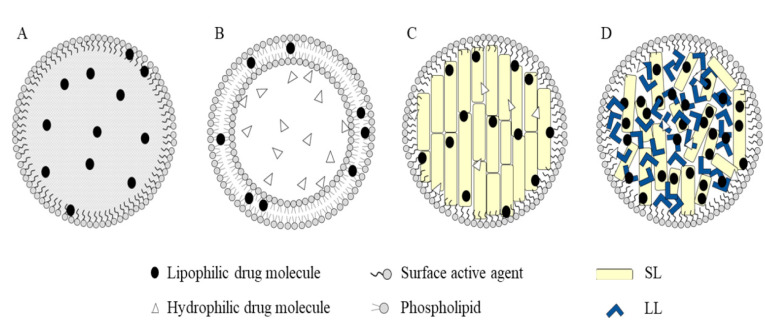
Different types of lipid-based nanoparticles: (**A**) nanoemulsions; (**B**) liposomes; (**C**) solid lipid nanoparticles (SLNs); (**D**) nanostructured lipid carriers (NLCs). Adapted from Haider et al. [67].

Lipid nanocarriers such as liposomes, solid lipid nanoparticles (SLNs), nanostructured lipid carriers (NLCs), and nanoemulsions (Figure 2) are considered an ideal strategy for effective delivery of the therapeutic agents against MS at the CNS, enhancing brain transport. They possess the ability to cross the BBB by naturally entering the brain capillary endothelial cells, reducing the peripheral side effects. Furthermore, through suitable surface decoration, lipid nanocarriers can be engineered to interact with particular types of molecules or cell receptors presented in the BBB, even delivering drugs which normally cannot cross the BBB [65,66].

#### 3.1.1. Nanolipid Carriers (NLCs) and Solid Lipid Nanoparticles (SLNs)

The lipid-based nanoparticulate system with a solid matrix primarily originated from an oil-in-water type emulsion, by replacing the oil phase or liquid lipids with solid lipids to make it solid at body temperature. Solid lipid nanoparticles (SLNs) are usually considered as the first-generation lipid nanoparticle developed from solid lipid, while nanolipid carriers (NLCs) are known as second-generation lipid nanoparticles, comprising a solid and liquid lipid blend, as well as a surfactant (Figure 2C,D) [68,69]. Although the formulation contains a liquid lipid, NLCs remain in the solid state at body and room temperatures, by adjusting the levels of the liquid lipid. SLNs are colloidal particles with increased physical stability derived from oil-in-water (O/W) emulsions, by replacing liquid lipids with a lipid matrix that is solid at both room and body temperature. The lipid core of SLNs typically consists of fatty acids, monoglycerides, diglycerides, triglycerides, waxes, or steroids and is stabilized by surfactants [70,71]. SLNs and NLCs differ in the composition and organization of the lipids of the matrix, which causes different morphological structures. Commonly used solid lipids for NLC formulation are stearic acid, stearyl alcohol, glycerol monostearate, mono-stearin, etc., while common examples of liquid lipids involve the use of olive oil, sesame oil, almond oil, peanut oil, soyabean oil, oleic acid, corn oil, soy lecithin, phosphatidyl choline, vitamin E, etc. [69].

Recently, Gadhave et al. [72] formulated intranasal nanolipid carriers loaded with TFM, an inhibitor of dihydroorotate dehydrogenase, exhibiting anti-inflammatory activity. The TFM-loaded NLCs were prepared via the melt emulsification ultrasonication method, while the Box–Behnken statistical design was applied to optimize the formulation. The lipid nanocarriers were composed of Compritol^®^ 888 ATO (solid lipid), maisine 35–1 (liquid lipid), gelucire 44/14 (stabilizer) (all by Gattefosse, Mumbai, India), and its aqueous phase from water and Tween-20 (surfactant) (S.D. Fine Chemicals, Pune, India). They were evaluated regarding their particle size, entrapment efficiency (%), in vitro and ex vivo permeation, and their pharmacological and toxicological properties. According to the results, the optimized formulation exhibited particle size, surface charge, and entrapment efficiency of 99.82 nm, −22.29 mV, and 83.39%, respectively. The formulation was enriched with mucoadhesive and gelling agents. The ex vivo drug permeation study and the permeation flux of the prepared mucoadhesive nanosystem was higher than the plain one. From the therapeutic point of view, the intranasal administration of the prepared nanostructures facilitates the rapid remyelination of damaged neurons in the cuprizone-treated rat model, without any significant change in hepatic biomarkers and sub-acute toxicity of the drug; thus, it can be considered as effective and safe delivery for brain disorders.

Kumar et al. [73] developed nanolipid carriers for the delivery of DMF and tocopherol acetate, in an effort to enhance the brain permeability of DMF, improve its gastric tolerance, and reduce its side effects. They used stearic acid (M/s Central Drug House, New Delhi, India) as the solid-phase lipid, tocopherol acetate as the liquid-phase lipid, and Tween 80 (M/s Fisher Scientific India Pvt. Limited, Mumbai, India) as an emulsifier, while the hot microemulsion technique was utilized as the preparation method. The prepared formulation was then evaluated by physicochemical, Caco-2 cellular permeability, in vitro drug release, in vivo pharmacokinetics, and biodistribution studies. The physicochemical study revealed characteristics suitable for brain drug delivery, namely, an average size of 69.70 nm, polydispersity index (PDI) of 0.317, and zeta potential of −9.71 mV. At the same time, the loading efficiency and entrapment efficiency of the formulation were observed as 20.13% and 90.12%, respectively. The obtained controlled release profile, in both gastric and intestinal pH (up to 68% of drug in 24 h), was attributed to the better encapsulation of DMF within the NLC. The drug release was found to best fit the Higuchi equation of release kinetics. Cellular uptake studies on Caco-2 and SH-SY5Y monolayers confirmed better intestinal absorption, due to its biodegradable, lipidic formulation and small size, as well as higher neuronal uptake of the developed system. Furthermore, the in vivo pharmacokinetic data showed a significant improvement in C_max_ and t_1/2_, three times higher drug absorption, and a reduction in the drug clearance, volume of distribution, T_max_, and drug elimination. The findings are promising and offer preclinical evidence for better brain bioavailability of DMF, which can improve the clinical therapy of MS through reduction of the dosing frequency.

Ghasemian et al. [74] developed baclofen-loaded nanolipid carriers for effective brain drug delivery, in order to reach the site of baclofen action in the CNS. Although baclofen is not an MS-specific therapy, it is used in the treatment of multiple sclerosis, in order to eliminate the spasticity that usually appears in MS. However, the effect of oral baclofen is limited due to its hydrophilic nature, which creates insufficient concentrations in the brain and CSF. The proposed lipid nanocarrier was prepared via the double emulsification solvent evaporation technique and was composed of glyceryl monostearate, glyceryl distearate, glyceryl trioleate (all by Gattefossè, France), and an aqueous phase containing Tween 80 (Sigma-Aldrich, Darmstadt, Germany), as a surface acting agent. Taking into account the obtained physicochemical results, the formulation gave suitable ranges of particle size, size distribution, and zeta potential, while encapsulation efficiencies ranged between 39% and 42%. Glyceryl trioleate lipid gave the minimum particle size. The obtained sustained release profile (up to 74.6% of drug in 28 h) was found to best fit the Higuchi equation of release kinetics and to take place via Fickian diffusion. The formulation of baclofen in nanolipid carriers increased the half-life of drug in plasma and brain by up to 10 and 1.5 times, respectively, and provided a prolonged effect compared to the solution formulation.

Most recently, Kumar et al. [75] loaded methylthioadenosine in SLNs for oral delivery to the brain for the management of MS. The SLNs were prepared via the well-reported microencapsulation technique and were composed of stearic acid (M/s Central Drug House, New Delhi, India), phospholipid 90 G (IPCA Laboratories, Mumbai, India), and Tween-80. The obtained SLNs exhibited sizes below 100 nm, being well within the range to offer a promise of enhanced BBB permeability and bypassing the reticuloendothelial system, with almost neutral zeta potential; they also offered higher drug entrapment and drug loading. Cuprizone-induced demyelination model in mice was employed to mimic the MS-like conditions of demyelination. We should mention that the cuprizone model does not accurately capture the MS disease state, but it is useful for pro-remyelination investigations. It is mainly neurodegenerative-based, exhibiting toxin-based demyelination, rather than immune-mediated demyelination [9]. The symptoms were monitored to some level by plain methylthioadenosine and to a major extent by the SLN version of this nucleoside. According to the pharmacokinetic studies, the methylthioadenosine was better absorbed from SLNs vis-à-vis plain methylthioadenosine, and the biological residence was substantially enhanced so as to reach the target site, thus improving bioavailability and enhancing bioresidence. Methylthioadenosine-loaded SLNs were able to maintain the normal metabolism, locomotor activity, motor coordination, balancing, and grip strength of the rodents compared to plain MTA. The pharmacokinetics corroborated the pharmacodynamic findings, indicating that the orally administrated SLNs can be substantially delivered to the brain and can effectively remyelinate the neurons.

In another case of SLNs, Gandomi et al. [76] developed polyethylene glycol (PEG)ylated SLNs in order to efficiently deliver the glycocorticosteroid methylprednisolone to the brain. The corticosteroids, including the glucocorticosteroids, which are mentioned in many literature cases of drug delivery nanosystems described in the present review article, are used in pulse curses, in order to clinically treat significant relapses in MS patients, in an attempt to hasten recovery. Although they are one of the most common clinically prescribed drugs for reducing MS symptoms, due to their anti-inflammatory and immunosuppressive effects, we should note that they are not a specific treatment of MS. The SLNs were surface-modified by using two targeting moieties and, more specifically, glycoprotein antigens, either anti-Contactin2 or anti-Neurofascin, which are two axo-glial-glycoprotein antigens located in the node of Ranvier and are considered to be the main targets of autoimmune reaction in MS. Myelin-based SLNs were prepared via the solvent evaporation method and were composed of myelin lipids including cholesterol, sphingosine, phosphatidylethanolamine, phosphatidylcholine, sphingomyelin, and phosphatidylserine (all by Sigma Aldrich, St. Louis, MO, USA). In order to obtain targeted SLNs, the antibody, either anti-Contactin2 or anti-Neurofascin, was conjugated to the drug-loaded polyethylene glycol (PEG)-covered SLNs (PEGylated SLNs). The prepared SLNs were physicochemically characterized, while their in vitro release profile, cell viability, and cell uptake were studied. Their brain uptakes were also probed following injections into MS-induced mice. The formulation differentiated the particle size; for example, smaller particle sizes following the antibody bindings were observed, due to the lesser hydration of polymer after the antibody conjugation. The SLNs presented good release profiles, where variations in the lipid type slightly affected the drug release profile, while the PEG and/or antibody coating provided a sink boundary condition for the drug diffusion within the lipoid matrix. It was found that the targeted PEGylated SLNs had no significant cytotoxicity on U87MG-cells, although their cellular uptake was increased four- and eight-fold when surface-modified with anti-Contactin2 or anti-Neurofascin, respectively, compared to control. However, the non-surface-modified SLNs exhibited better penetration ability of the BBB compared to the PEGylated and antibody-functionalized SLNs. The authors suggested that the antibodies may facilitate the adsorption of the SLNs to myelin due to binding to the related antigens, i.e., Contactin2 or Neurofascin, on the CNS axon, and the above information would help toward the development of more efficient nanocarriers for the treatment of MS.

#### 3.1.2. Liposomes

Liposomes (Figure 2B) are considered to be one of the most well-investigated drug delivery nanosystems, presenting major advantages, such as biocompatibility and biodegradability, while also exhibiting great versatility, because they can be easily surface-modified with functional biomaterials, in order to acquire advanced properties, such as escaping rapid clearance in circulation and presenting increased targeting to pathological tissues [54,77].

In addition to lipid nanoparticles, liposomes and especially the PEGylated ones were also reported as potential drug carriers for MS and specially for the delivery of glycosteroids. Schmidt et al. [78] developed a novel formulation of PEG-coated long-circulating liposomes, prepared from dipalmitoyl phosphatidylcholine (Lipoid GmbH, Ludwigshafen, Germany), encapsulating prednisolone that was administrated to the CNS of rats exhibiting EAE. The authors tried to achieve ultra-high tissue concentrations of glucocorticosteroids in the inflamed target organ as compared to an equivalent dose given as free drug, along with a much lower systemic concentration with a reduction of unwanted side effects. Radioactive labeling showed the accumulation of liposomes in the inflamed target organ. More specifically, ^3^H-labeled prednisolone liposomes showed selective targeting to the inflamed CNS, where up to 4.5-fold higher radioactivity was achieved compared to healthy control animals. Moreover, much higher and more persistent levels of prednisolone in the spinal cord were detected by liposomal administration than in the case of free administrated drug. Gold-labeled liposomes were used, while they could be detected in the spinal cord within the vascular endothelium, as well as in inflammatory macrophages, microglial cells, and astrocytes. The BBB disruption, the T-cell and macrophage infiltration, and the percentage of tumor necrosis factor-α (TNF-α) in these cells were monitored, indicating superior performance by the liposomal administration of prednisolone. It was also reported that a single injection of the liposomes clearly ameliorated the course of adoptive transfer EAE and EAE induced by immunization. The authors finally stated that the liposomal prednisolone was found to be highly effective in the treatment of EAE, being superior to a five-fold higher dose of free methylprednisolone, possibly due to liposomal targeting.

Later, Gailard et al. [79] used PEGylated liposomes conjugated to the brain-targeting ligand glutathione (GSH-PEG), in order to optimally improve the therapeutic window of methylprednisolone, composed of hydro soy phosphatidylcholine lipid (Lipoid, Cham, Switzerland). The authors chose the GSH-PEG liposomes because they include the safety of the liposomal constituents, the ability to encapsulate compounds without modification, the prolonged plasma exposure, and the possibility to enhance drug delivery to the brain. The prepared liposomes were administrated to rats with acute EAE. Apart from the prolonged plasma circulation and increased brain uptake as revealed by the pharmacokinetic analysis, the treatment with GSH-PEG liposomes was found to be significantly more effective in EAE as compared to PEG liposomes, while the same dose level of free methylprednisolone even worsened disease outcome. The rats received intravenous treatment, before disease onset, at disease onset, or at the peak of disease. Free methylprednisolone and non-targeted pegylated (PEG) liposomal methylprednisolone served as control treatments. It was reported that, when the treatment was initiated at disease onset, free methylprednisolone showed no effect, while GSH-PEG liposomal methylprednisolone significantly reduced the clinical signs to 42% ± 6.4% of the saline control, confirming that GSH-PEG liposomes improve the therapeutic availability of methylprednisolone and, therefore, a lower dose and lower dosing frequency can be used to obtain an effective brain concentration.

Lee et al. [80] further exploited the efficacy of the GSH-PEG liposomes carrying methylprednisolone, by using mice exhibiting murine myelin oligodendrocyte-induced EAE (MOG-EAE). This animal model mimics many neurodegenerative features of MS, including axonal damage. The experimental protocol was as follows: after the disease onset, mice were randomized to receive saline, three injections of free drug (high dose methylprednisolone), two injections of free drug (low dose methylprednisolone), or two injections of liposomes. The infiltration of T cells and macrophage/microglia, the amount of astrocyte activation, the extent of axonal loss, and the demyelination in spinal cord lesions were also monitored, indicating good performance of liposomes compared to a low dose of the free drug. Treatment with a low dose of liposomes significantly reduced the severity of EAE, similar to treatment with high-dose free drug but at one-tenth of the dosage, while a low dose of free methylprednisolone was not effective. The proposed liposomes were clinically and histologically effective as a high dose of free drug, thus allowing treatment by liposomal administration at a lower application frequency.

More recently, sterically stabilized liposomes were designed by Turjeman et al. [81] in order to carry glucocorticosteroids and treat the neuroinflammation presented in MS. More specifically, the authors investigated the remote loading of the “water-soluble”, amphipathic weak acid glucocorticosteroid prodrug methylprednisolone hemisuccinate (MPS) or the amphipathic weak base nitroxide tempamine (TMN) and compared the effect of passive targeting alone and of active targeting based on short peptide fragments of ApoE or of β-amyloid. The stealthiness of the liposomes was achieved by incorporating the PEG-DSPE-2000 lipid (Genzyme Pharmaceuticals, Liestal, Switzerland). The peptide-conjugated sterically stabilized liposomes (actively targeted) were prepared via the covalent attachment of either ApoE or β-amyloid to dioleoyl (DO)-succinate, in order to form the lipidated peptides. These two peptides can be transported through the BBB. In addition to the physicochemical and thermotropic characterization of the fabricated liposomes, the EAE mice model was used, in order to monitor their therapeutic efficacy, while its mechanism of action in both the acute and the adoptive transfer EAE models was investigated. According to the results, for the liposomes carrying MPS, active targeting is not superior to passive targeting. For both groups of liposomes, carrying MPS or TMN, it was demonstrated that these nano-drugs ameliorated the clinical signs and the pathology of EAE. The authors concluded that the highly efficacious anti-inflammatory therapeutic feature of these two nano-drugs meets the criteria of disease-modifying drugs and supports further development and evaluation of these nano-drugs as potential therapeutic agents for diseases with an inflammatory component.

The prevalence of autoimmunity is on the rise, and there is no cure for any autoimmune disease, caused by the loss of tolerance to self. Apart from delivering drugs, liposomes were applied as immunotherapy, due to their ability of apoptosis to induce immunological tolerance. One of the mechanisms to maintain self-tolerance is the efficient removal of apoptotic cells. For example, liposomes were generated mimicking apoptotic β-cells, where they arrest autoimmunity in type 1 diabetes, through specific and definitive re-establishment of tolerance. This type of liposome was also investigated to be applied in other autoimmune diseases, such as MS. More analytically, phosphatidylserine-rich liposomes were prepared and loaded with MS-specific autoantigen and, more specifically, the myelin-oligodendrocyte glycoprotein peptide 40–55 (MOG_40–55_), in order to co-deliver a double signal of tolerance and specificity to arrest autoimmunity in a synergistic, effective, and safe manner. Phosphatidylserine is considered to be the main “eat me” and “tolerate me” signal of the apoptotic cell membrane, which allows recognition and phagocytosis by antigen-presenting cells, such as dendritic cells. According to the results, the prepared liposomes induced a tolerogenic phenotype in dendritic cells and arrested autoimmunity, while they were efficiently phagocytosed by dendritic cells and induced tolerogenic features in dendritic cells. Moreover, after immunization administration, the MOG-loaded phosphatidylserine liposomes reduced the incidence and severity of EAE, as well as delayed the onset of the disease, correlating with an increase in the regulatory CD25+ FoxP3− CD4+ T-cell subset. The authors concluded that the proposed nanosystems exhibit high potential to operate as a platform for MS and autoimmune diseases [82].

#### 3.1.3. Other Lipid-Based Nanocarriers

Binyamin et al. [83] developed a nanodroplet formulation of pomegranate seed oil (PSO), denominated as nano-PSO, which was an oil-in-water (O/W) nanoemulsion (Figure 2A) administrated in an EAE model. PSO comprises high levels of punicic acid, a unique poly-unsaturated fatty acid considered as one of the strongest natural antioxidants. Nano-PSO was supposed to enhance the bioavailability and activity of PSO. According to the results, the beneficial effect of PSO was increased significantly when EAE mice were treated with nano-PSO of specific size nanodroplets (200 nm) size and much lower concentrations of the oil. Nano-PSO was also beneficial to the EAE mice when treatment commenced close to disease manifestation (day 7) and not only when administered concomitant with disease induction, highlighting its potential to abrogate the disease progression and not only prevent it. Pathological examinations revealed that nano-PSO administration dramatically reduced demyelination and oxidation of lipids in the brains of the affected animals, even in the existence of immune infiltrates in the CNS. Nano-PSO may be a good choice for individuals at the initial stages of MS, as well as at later stages, where it may be used in combination with advanced MS treatments such as natalizumab or antioxidant formulations. Last but not least, nano-PSO was also beneficial in the prevention and treatment of genetic prion disease model, indicating that reagents that can prevent lipid oxidation may be beneficial for an array of neurodegenerative diseases.

Most recently, Lu et al. [84] tried to take advantage of the significant role of the monocytes in the process of MS and, thus, developed a targeting immunomodulatory carrier from high-density lipoprotein-mimicking peptide–phospholipid scaffold (HPPS), which can target monocytes, in order to improve the bioavailability of curcumin. Monocytes are considered to be mediators and immunomodulation targets because they are considered to be similar to most of the amplified inflammatory monocytes crossing the BBB, to promote neuron injury and recruit more immune cells to infiltrate the CNS. More specifically, peripheral monocytes were chosen as the immunomodulation targets and curcumin as the anti-inflammatory agent, which was delivered by HPPS nanoparticles. The nanoparticles were taken up efficiently, specifically by monocytes through the scavenger receptor class B type I (SR-B1) receptor, which is a receptor of high-density lipoprotein (HDL) that is highly expressed in the peripheral monocytes; thus, the proposed nanoparticles could be used for early detection of CNS inflammation in EAE. The nanoparticle distribution in monocytes was confirmed in vivo by using optical imaging, while the nanoparticles were loaded with a fluorescent dye. According to the results, the delivery of curcumin by HPPS nanoparticles hindered inflammatory monocytes across the BBB in EAE mice, owing to the downregulation of intercellular adhesion molecules 1 (ICAM-1) and macrophage-1 antigen (MAC-1) expression in the monocytes by inhibiting the activation of the nuclear factor-κB (NF-κB). Moreover, the proposed nanoparticles inhibited the proliferation of microglia and restricted the infiltration of other effect or immune cells, such as TH1, TH17, and myeloid cells, due to the blockade of inflammatory monocyte infiltration, resulting in the reduction of EAE morbidity from 100% (10 of 10 mice exhibited EAE pathology in the phosphate-buffered saline (PBS) group) to 30% (three of 10 mice showed mild clinical signs). The authors concluded that the targeted modulation of monocytes with such HPPS nanoparticles, carrying therapeutic and/or imaging agents, offers a novel strategy for MS diagnosis and treatment.

MicroRNAs were postulated as a promising tool to induce OPC differentiation and, therefore, remyelination, albeit exhibiting significant limitations in terms of the administration of microRNAs to the CNS. Osorio-Querejeta et al. [85] utilized three different categories of nanosystems for miR-219a-5p encapsulation, release, and remyelination promotion, namely, liposomes from 1,2-dioctadecanoyl-sn-glycero-3-phosphocholine (DSPC) lipid (Avanti Polar Lipids, Alabaster, AL, USA), poly(lactic-*co*-glycolic) acid polymeric nanoparticles, and finally biologically engineered extracellular vesicles overexpressing miR-219a-5p, which are also lipid-based, due to their biological origin. Extracellular vesicles are biological delivery systems that contain other proteins, lipids, and genetic material, apart from RNA, which can be integrated into the cell in several ways, and which contain microRNA-processing molecules. The three nanosystem categories were compared by assessing their ability to induce OPC differentiation in a primary oligodendrocyte precursor cell culture and cross the BBB. According to the results, on the one hand, the liposomes and the polymeric nanoparticles were able to entrap higher amounts of miR-219a-5p and showed higher uptake levels than extracellular vesicles. On the other hand, the extracellular vesicles, due to their biological complexity, were surprisingly the only delivery system that was able to induce a significant OPC differentiation, while they also showed the highest BBB permeability levels. Finally, the EAE animal model was used to study the remyelination potential of the extracellular vesicles. The intranasally administered miR-219a-5p-enriched extracellular vesicles successfully decreased clinical scores in the EAE model, without affecting the tested anti-inflammatory pathways. As the authors concluded, the significant differences in clinical score observed after the disease peak indicate that extracellular vesicles might be increasing the myelin production.

### 3.2. Polymer-Based Nanosystems

Several types of polymers and their respective nanoparticles were studied for efficient BBB crossing, the delivery of therapeutic agents to the CNS, and their contribution to the treatment of neurodegenaration diseases, such as MS. Polymer nanoparticles can be prepared using various synthetic and natural monomers/polymers and via different preparation methods. Their surface can also be functionalized for specific brain targeting. It is crucial for the used polymers in manufacturing these nanocarriers to be biocompatible and biodegradable. The mechanisms for brain uptake and drug release to the CNS of the polymeric nanoparticles involve endocytosis or transcytosis through the endothelial cells, as well as accumulation in the brain capillaries, resulting in transfer to the brain parenchyma, owing to the high concentration gradient, and membrane fluidization through lipid solubilization, due to the surfactant effect and tight junctions opening. One of their most significant advantage is their potential to be easily surface-functionalized by the conjugation of targeting peptides or cell-penetrating ligands, in order to improve their targeting ability and crossing through the BBB [86].

#### 3.2.1. Poly(Lactic-*co*-Glycolic Acid) (PLGA) Polymeric Nanoparticles

Polymeric poly(lactic-*co*-glycolic acid) (PLGA) or poly(lactide-*co*-glycolide) (PLG) nanoparticles are attractive carriers due to their biodegradability, bio-compatibility, and approval by the FDA. One of the key advantages of using PGLA nanoparticles is that they can be easily loaded with a wide variety of molecules. The degradation kinetics of nanoparticles and the release rate of the encapsulated molecules can be easily monitored by controlling the PGLA physicochemical properties, such as the lactide-to-glycolide ratio, molecular weight, crystal profile, storage temperature, and surface coating materials [87,88,89,90,91].

**Figure 3:**
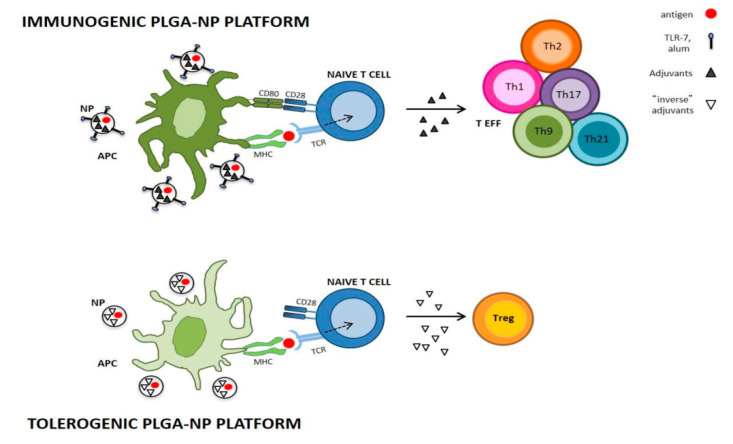
Immunogenic and tolerogenic poly(lactic-*co*-glycolic acid) (PLGA) NP (nanoparticle) platforms. Immunogenic PLGA-NPs may include standard adjuvants such as alum or Toll-like receptor 7 (TLR-7) agonists, capable of activating APCs (antigen-presenting cells) and promoting naïve T-cell differentiation into effector T cells (TEFFs); on the contrary, in the absence of costimulatory signals, tolerogenic PLGA-NPs by employing “inverse adjuvants” induce tolerogenic APCs that lead to the expansion of antigen-specific regulatory T cells (Tregs). The dotted arrow indicates activation of naïve T cells, and the solid arrow indicates its differentiation into TEFFs or Tregs. Adapted from Cappellano et al. [94].

“Inverse vaccination” includes antigen-specific tolerogenic immunization treatments that are capable of inhibiting autoimmune responses, exhibiting great therapeutic potential in MS. Antigen-specific treatments are highly desirable for autoimmune diseases in contrast to treatments which induce systemic immunosuppression. Several myelin proteins, such as MBP, PLP, and MOG, were implicated as targets of autoreactive T cells in MS and EAE [92]. The use of protein-based inverse vaccines in the EAE model, loaded in polymeric biodegradable PLGA nanoparticles, in order to obtain the sustained release of antigens and regulatory adjuvants, can be an alternative strategy to overcome the main obstacles of the administration of free myelin antigens, which exhibit rapid clearance, or of the DNA-based vaccines encoding for myelin autoantigens, whose potential risks limit their use in humans. These data suggest that subcutaneous PLGA nanoparticle-based inverse vaccination maybe an effective tool to treat autoimmune diseases (Figure 3), such as MS, and reduce the significant toxicity, as well as the high costs, caused by the chronic therapies [93,94].

Cappellano et al. [93] developed PLGA nanoparticles loaded with either the immunodominant 35–55 epitope of MOG (MOG_35–55_) in C57BL/6 mice or the recombinant interleukin-10 (IL-10), used as an inverse adjuvant, for prophylactic and therapeutic treatment of a chronic progressive model of EAE. The authors prepared 65:35 PLGA nanoparticles because they slowly release the loaded molecule for several weeks, which was also confirmed in vitro, and they display minimal cell toxicity along with low intrinsic adjuvant activity. More specifically, the prepared nanoparticles did not display cytotoxic or proinflammatory activity and were partially endocytosed by phagocytes. The prepared nanoparticles loaded with IL-10 completely lost their ability to induce secretion of TNF-α in vitro in peripheral blood mononuclear cells, while the in vivo subcutaneous injection of the prepared nanoparticles in C57BL/6 mice, loaded with either MOG_35–55_ or IL-10, being both simultaneously injected, ameliorated the course of EAE in both prophylactic and therapeutic vaccination. The EAE onset was earlier in the prophylactic vaccination than in the therapeutic vaccination, which suggests that pre-treatment with the PLGA nanoparticles may precondition the induction of EAE by stimulating the innate immunity. Contrariwise, immunization with only one type of nanoparticle, loaded with either MOG_35–55_ or IL-10, did not have any effect. Moreover, they decreased the histopathological lesions in the central nervous tissue, the inflammation, and the T-cell infiltration in the CNS, as well as the secretion of proinflammatory cytokines IL-17 and interferon (IFN)-γ induced by MOG_35–55_ in splenic T cells in vitro.

Maldonaldo et al. [95] developed synthetic, biodegradable PLGA nanoparticles loaded with the immunodominant 139–151 epitope of myelin proteolipid protein (PLP_139–151_) together with the tolerogenic immunomodulator rapamycin, used as an inverse adjuvant, in order to induce durable and antigen-specific immune tolerance, even in the presence of potent Toll-like receptor agonists and control both cellular and humoral immune responses. The nanoparticles were administrated in a relapse remitting model of EAE in mice. According to the results, the treatment, by either subcutaneous or intravenous administration, with tolerogenic nanoparticles results in the inhibition of the activation of the antigen-specific CD4+ and CD8+ T cell, an increase in regulatory cells, durable B-cell tolerance with resistance to multiple immunogenic challenges, and the inhibition of antigen-specific hypersensitivity reactions at relapsing EAE. More specifically, when Swiss Jack Lambert (SJL) mice were immunized with the PLP_139–151_ peptide in complete Freud’s adjuvant (PLP_139– 151_/CFA) and treated therapeutically with a single dose of tolerogenic nanoparticles at the peak of disease, the mice were completely protected from developing relapsing paralysis. Prophylactic treatment using these nanoparticles inhibited the onset of EAE, whereas the therapeutic treatment inhibited relapse. Moreover, an inhibition of the neutralizing antibody responses against coagulation factor VIII in hemophilia A mice, was observed, even in animals previously sensitized to antigen. Only the encapsulated rapamycin and not the free form in solution could induce immunological tolerance, preventing both cellular and humoral immunity. The authors concluded that therapy with tolerogenic nanoparticles can be applied against allergies and autoimmune diseases, such as MS, as well as for the prevention of antidrug antibodies against biologic therapies.

Cho et al. [96] developed a formulation of dual-sized, polymeric microparticles from PLGA, referred as dMPs, which were loaded with multiple immunomodulatory factors, namely, specific antigen and tolerizing factors, targeting both intra- and extracellular tolerogenic receptors, in order to block the autoimmunity. The epitope MOG_35–55_, along with vitamin D, was encapsulated in the phagocytosable small particles, while transforming growth factor beta 1 (TGF-β1) and granulocyte-macrophage colony-stimulating factor (GM-CSF) were encapsulated in the large non-phagocytosable particles. According to the results, the administration of the dMPs resulted in a reduction of infiltrating CD4+ T cells, inflammatory cytokine-producing pathogenic CD4+ T cells, activated macrophages, and microglia in the CNS, as well as reduced frequency of CD86^hi^MHCII^hi^ dendritic cells in draining lymph nodes of EAE mice. Thus, the formulation was successfully recruited and modulated the dendritic cells toward a tolerogenic phenotype, while also exhibiting a local controlled release and achieving robust durable antigen-specific autoimmune protection.

Tolerogenic PLGA nanoparticles can also function as on-target and direct modulators of myelin-autoreactive T cells without eliciting the intervention of tolerogenic dendritic cells. Pei et al. [97] developed PLGA nanoparticles for the encapsulation of multiple regulatory molecules. More specifically, the TGF-β was encapsulated in nanoparticles that were surface-decorated by multimers of MHC class I and II molecules loaded with myelin peptides to target autoreactive T cells (MOG_40–54_/H-2D^b^-Ig dimer, MOG_35–55_/I-A^b^ multimer), by the regulatory molecules (anti-Fas, PD-L1-Fc), being capable of inducing apoptosis or dysfunction of the autoreactive T cells bound to the MHC multimers, or by the “self-marker” CD47-Fc, being able to inhibit nanoparticle phagocytosis. The suggested nanoparticles exhibited a size of 217 nm and were administrated by intravenous infusion, where they were capable of durably ameliorating EAE, with a marked reduction of clinical score, neuroinflammation, and demyelination. Their mechanism of action was based on the inhibition of the myelin-autoreactive T-cell surface presentation of multiple ligands and the paracrine release of cytokine. According to the results, the MOG_35–55_-reactive Th1 and Th17 cells, as well as the MOG_40–55_-reactive Tc1 and Tc17 cells, were decreased, and the regulatory T cells were increased, while inhibited T-cell proliferation and elevated T-cell apoptosis in the spleen took place.

Another report on inducing peripheral, antigen-specific T-cell tolerance for treatment of MS describes the use of biodegradable PLG tolerogenic nanoparticles, being loaded with myelin antigens (PLP_139–151_ and OVA_323–339_ (ovalbumin)) and administrated in mice with relapsing/remitting EAE. PLG particles were fabricated on-site via an emulsion process with modifications using poly(ethylene-*co*-maleic acid) as a surfactant (PLG-PEMA), in order to increase their ability to couple with peptides and to prevent disease induction. In addition to the characterization of the physical properties of the particles, their safety and their therapeutic efficacy for EAE were evaluated by clinical score, histology, and flow cytometric assessment of CNS inflammatory cell infiltration. Their intravenous infusion yielded a significant improvement in the ongoing disease and subsequent relapses when administered at onset or at peak of acute disease, as well as a minimization of epitope spreading when administered during disease remission, resulting in complete long-term protection from disease with superior effects to the already existing commercial PLG nanoparticles. The tolerance was induced by the combined effects of T-cell anergy and the activation of Treg. According to the results, there were reduced CNS infiltration of encephalitogenic Th1 (IFN-γ) and Th17 (IL-17a) cells, as well as inflammatory monocytes/macrophages and demyelination in the CNS of the treated animals [98].

Casey et al. [99] compared the differences between the intravenous and subcutaneous administration of PLG nanoparticles containing disease-relevant antigens (denoted as silver nanoparticles (Ag-NPs)), being surface decorated with TGF-β. The purpose of Ag-NP therapies is to treat autoimmunity. Although the Ag-NPs being intravenously administrated exhibit antigen (Ag)-specific immune tolerance in models of autoimmunity, there is a lower efficacy with subcutaneous administration. Thus, the authors tried to investigate whether the co-delivery of the immunomodulatory cytokine TGF-β using the Ag-NPs would modulate the immune response to Ag-NPs and improve the efficiency of tolerance induction. The selected antigen was PLP_139–151._ An in vitro co-culture system of bone marrow-derived dendritic cells (BMDCs) and naive T cells was used to evaluate the bioactivity and the immunomodulatory effects of the Ag-NPs with TGF-β, by monitoring the surface costimulatory markers and inflammatory cytokine production. According to the results, the Ag-NPs with TGF-β provided Ag-specific T-cell stimulation, decreased co-stimulatory molecule presentation, and suppressed inflammatory cytokine secretions. The in vivo Treg frequency and number were measured following the injection of particles into OT-II mice that expressed OVA_323–339_-restricted T-cell receptors, resulting in the surface binding of TGF-β to PLGA-NPs loaded with OVA being required to efficiently induce tolerance to OVA in an antigen-specific manner. Finally, the ability of surface-bound TGF-β to enhance the tolerogenicity of Ag-NPs was evaluated using the EAE mouse model in the context of intravenous and subcutaneous administration routes, revealing improved efficacy at lower doses by intravenous administration and significantly reduced disease severity by subcutaneous administration. Thus, subcutaneous inverse vaccination needs inverse adjuvants in order to be effective.

Getts et al. [100] used either polystyrene or biodegradable PLG microparticles bearing encephalitogenic peptides, being administrated by intravenous infusion, in order to prevent the onset and modify the course of the disease. These antigen-decorated microparticles, exhibiting an approximate 500-nm diameter induced a long-term T-cell tolerance in mice with relapsing EAE. The chosen antigen for the nanoparticle decoration was MOG_35–55_, being covalently linked to the surface of the nanoparticle. This treatment reduced the inflammatory cell infiltration and the damage of the CNS. The authors stated that the beneficial effect of these antigen-linked particles requires the scavenger receptor MARCO, being expressed at the marginal zone macrophages. Moreover, the results of the treatment were also monitored by the activity of regulatory T cells, the abortive T-cell activation, and the T-cell anergy. The obtained results highlighted the potential for using microparticles in order to target natural apoptotic clearance pathways, to inactivate pathogenic T cells, to stop the disease process in autoimmunity, and to treat T-cell-based autoimmune disorders, by inducing T-cell tolerance.

Kuo et al. [101] investigated the effect of the amount of antigen conjugated to PLG nanoparticles, as well as of the nanoparticle dose for the induction of immune tolerance, during the treatment of MS with disease-relevant antigens. More specifically, different amounts of the PLP_139–151_ antigen were loaded in PLG nanoparticles that were intravenously administrated in vivo, in different doses to mice with EAE. As a result, the amounts of antigen conjugation and nanoparticle dose were correlated with the severity of EAE. More specifically, the high dose of PLG nanoparticles carrying high amounts of PLP_139–151_ significantly decreased the severity of the EAE with a more durable immune tolerance, also preventing relapses, while a low dose of the nanoparticles carrying high amounts of the antigen or a high dose of the nanoparticles carrying low antigen amounts were not so efficient, as indicated by the observed relapses. The increase of nanoparticle dose and antigen amount was also correlated with the suppression of inflammatory signaling pathways in vitro. Through the analysis of the cells expressing MHC-restricted antigen, significant decreases in positive co-stimulatory molecules (CD86, CD80, and CD40) and a high expression of a negative co-stimulatory molecule (PD-L1) were found in high doses of both nanoparticles and with high antigen conjugation. Tolerance induction was evaluated by co-culturing cells administrated with the nanoparticles and autoreactive T cells isolated from mice immunized against PLP_139–151_. According to the results, reduced T-cell proliferation, increased T-cell apoptosis, and a stronger anti-inflammatory response were observed.

More recently, Saito et al. [102] investigated whether the use of multiple nanoparticle formulations and the extent of antigen loading at the carrier could impact the immune cell internalization and polarization of the immune cells that get associated with the particles and the subsequent disease progression. The nanoparticles were administrated for antigen-specific immunotherapy for the efficient induction of tolerance in the EAE disease model. More specifically, the formulation was composed of three polymeric carriers, 50:50 poly (dl-lactide-*co*-glycolide) with inherent viscosity (IV) = 0.17 dL/g and 0.66 dL/g (termed agPLG-L and agPLG-H, respectively), and poly(dl-lactide) (PLA) particles with IV = 0.21 dL/g (termed agPLA) loaded with the disease-specific antigen myelin protein (PLP_139–151_), being administrated in a single injection for mice with EAE. According to the results, at a low particle dose, mice treated with PLA-based particles had significantly lower clinical scores at the chronic stage, while neither PLG-based particles nor OVA control particles reduced the clinical scores. Moreover, the higher antigen loading PLA-based particles helped to reduce inflammation during the acute stage of the disease, as well as completely ameliorate EAE over 200 days, along with the inhibition of Th1 and Th17 polarization, allowing a smaller particle dosage and, thus, fewer potential adverse effects. PLA, which was correlated with a more tolerogenic polarization of the antigen-presenting cells, was found to be a more efficacious platform relative to PLG. Fluorescently labeled particles were employed to examine the biodistributions among the organs, the interaction with specific antigen-presenting cells within the organs, and the resulting phenotypes. Compared to PLG-based particles, PLA-based particles, interacting with antigen-presenting cells, were largely associated with Kupffer cells and liver sinusoidal endothelial cells, reducing the CD4+ T-cell populations that were activated locally and not trafficked in the CNS.

In addition to antigen-specific tolerogenic immunization, the PLGA nanoparticles were also employed in the remyelination strategy. Rittchen et al. [103] developed a PLGA nanoparticle-based strategy for the targeted delivery of leukemia inhibitory factor (LIF) to OPCs, in order to promote their differentiation into mature oligodendrocytes that are able to repair myelin. Among the potential therapeutics that promote remyelination is the LIF, a cytokine known to play a key regulatory role in self-tolerant immunity and recently identified as a promyelination factor. However, LIF is rapidly degraded in vivo, and high doses may have unwanted off-target effects; therefore, a nanotechnology strategy could help toward its targeted delivery to OPCs. In detail, PLGA-based nanoparticles of ~120 nm diameter were prepared, loaded with LIF (LIF-NP), and functionalized with surface antibodies against NG-2 chondroitin sulfate proteoglycan, expressed on OPCs. The nanoparticle platform used in this study previously featured in clinical trials (e.g., Clinicaltrials.gov NCT01812746, NCT01792479). According to the in vitro results, the NG2-targeted LIF-NPs bound to OPCs activated pSTAT-3 signaling and induced OPC differentiation into mature oligodendrocytes. As per the in vivo results, where a model of focal CNS demyelination was used, the NG2-targeted LIF-NP increased myelin repair, at the level of both increased number of myelinated axons and increased thickness (maturity of sheath) of myelin per axon. The authors also noted that the potency was high, because even a single dose of nanoparticles delivering picomolar quantities of LIF was sufficient to increase remyelination. The nanoparticles were added intralesionally, as a proof of concept, confirming the powerful potential of this delivery system as a targeted approach to deliver bioactive molecules for in situ myelin repair. The authors concluded that, for translation into clinical use, it is notable that the intravenously delivered nanoparticles should be able to cross the BBB, at least in rodents, or they can be delivered intranasally directly to the CNS, bypassing the BBB.

#### 3.2.2. Other Polymeric Nanoparticles

Führmann et al. [104] tried to exploit the physiopathology of MS, regarding the presence of leaky permeable blood vessels, where fibrinogen and nidogen are progressively upregulated after disease onset. Peptide-modified polymeric nanoparticles that are able to target the blood clots and the extracellular matrix (ECM) molecules, such as nidogen, can be used for the targeted drug delivery and the reduction of “off-target” effects. More specifically, poly(ethylene glycol)-*block*-poly(caprolactone) (PEG-*b*-PCL) nanoparticles, functionalized with fibrin peptides, were developed. The prepared nanoparticles were administrated in rats that exhibited EAE along with upregulation of fibrin and nidogen/entactin-1. The administrated nanoparticles showed enhanced binding to these targets and to lesion sites ex vivo and in vivo, compared to non-functionalized or scrambled-peptide control nanoparticles. By using a minimally invasive technique, the active targeting of leaky blood vessels in the diseased spinal cord of animals with EAE can be achieved after the systemic injection of fibrin-targeting nanoparticles. Thus, increased drug concentrations at the site of injury, reduction of undesirable side effects of systemic delivery, and enhanced efficacy of hydrophobic drugs can be achieved in MS conditions.

In another study [105] employing PCL nanoparticles, the recombinant human myelin basic protein (rhMBP) was purified from the milk of transgenic cows, by using a vacuum-driven cation exchanger, and it was formulated into PCL nanoparticles in order to achieve an rhMBP controlled release kinetic, fabricating a therapeutic vaccine for protection against EAE symptoms in mice. RhMBP-loaded PCL nanoparticles were prepared and characterized in terms of entrapment efficiency, size, morphology, charge, and release pattern. The nanoparticles were optimized, until discrete spherical, rough-surfaced rhMBP nanoparticles with small particle size, high surface charge, lower concentration of stabilizer, and intermediate concentration of polymer were obtained, in order to achieve high entrapment efficiency and a controlled release pattern. According to the in vivo results, after the subcutaneous administration of free or rhMBP nanoparticles before EAE induction, the average behavioral score in EAE mice was reduced, and only mild histological alterations and preservation of myelin sheath were observed, indicating increased protection and enhanced efficacy of rhMBP as a therapeutic vaccine due to the nanoformulation. Being hydrophobic, the PCL nanoparticles provided an improved drug delivery system for rhMBP, enabling it to cross the BBB and deliver rhMBP into the mice brains, while the differences in activity between the immediate release and sustained release of the nanoparticle formulations were highlighted. Moreover, the analysis of inflammatory cytokines (IFN-γ and IL-10) in mice brains revealed that the pre-treatment with free or rhMBP nanoparticles significantly protected against EAE-induced behavioral, histopathological, and inflammatory changes.

Lunin et al. [106] developed poly(butylcyanoacrylate) (PBCA) nanoparticles, in order to deliver the thymic peptide thymulin and prolong its presence in the blood of mice with relapsing/remitting EAE (rEAE). The increase in the blood content of thymulin, which is depleted with age, and the prolongation of its blood half-life, alone or in combination with other treatments, may be a prospective strategy for treatment of chronic inflammatory conditions, such as MS. More analytically, as revealed by the results, thymulin significantly decreased symptoms of rEAE and lowered plasma cytokine levels, both in early and in later stages of rEAE, as well as decreased the NF-κB and stress-activated protein kinase/Jun amino-terminal kinase (SAPK/JNK) cascade activation, as confirmed by ELISA measurements of cytokine levels in the blood. In terms of the cytokine response in rEAE, it was multi-staged, where an early phase was accompanied by an increase in plasma IFN-γ, while the IL-17 response was markedly increased at a later stage. According to the results, the nanoparticle-bound thymulin had enhanced efficacy in comparison to the effect of free thymulin.

Kondiah et al. [107] synthesized a pH-sensitive copolymer (TMC-PEGDMA-MAA) from trimethyl-chitosan (TMC), poly(ethylene glycol)dimethacrylate (PEGDMA), and methacrylic acid (MAA), via free radical suspension polymerization, in order to produce microparticles for the oral delivery of IFN-β. The polymer was designed to possess pH-sensitive, mucoadhesive, and hydrophilic properties. A Box–Behnken experimental design was used for optimization of the formulation, where varying concentrations of TMC and percentage crosslinker (polyethylene glycol diacrylate) were investigated. The prepared copolymeric microparticulate system was characterized for its morphological, porositometric, and mucoadhesive properties. The optimized microparticles with 0.5 g/100 mL TMC and 3% crosslinker had an IFN-β loading efficiency of 53.25%. The microparticles were subsequently compressed into a suitable oral tablet formulation. The in vitro release of IFN-β had a pH-sensitive pattern, being increased in intestinal (pH 6.8) and decreased in gastric (pH 1.2) environments. Regarding the in vivo results, the tablets were orally administrated in the New Zealand White rabbit, and the plasma concentration of IFN-β was compared to a known subcutaneous formulation during a 24-h blood sampling procedure. The IFN-β-loaded particulate system demonstrated a remarkable drug release in vivo, greater than the subcutaneous commercial formulation (Rebif^®^) over a 24-h duration, with a larger bioavailable IFN-β concentration after 2 h.

Youssef et al. [108] investigated the effect of intranasal administration of LINGO-1-directed small interfering RNA (siRNA)-loaded chitosan nanoparticles on demyelination and remyelination processes in a rat model of demyelination. Chitosan nanoparticles are one of the most studied polymers in non-viral siRNA delivery, due to their polycationic nature and biocompatibility. Suppression of LINGO-1 by different strategies, such as LINGO-1 gene knockout or infusion of LINGO-1 antagonists, was associated with enhancement of remyelination in different animal models of CNS demyelination. The RNA interference is a new strategy to block LINGO-1 expression in the target lesions with low doses and fewer systemic side effects, provided that there are efficient gene delivery carriers. In detail, the authors studied whether the nasal administration of LINGO-1-directed siRNA-loaded chitosan nanoparticles in rats could inhibit pontine LINGO-1 expression and enhance the remyelination in a model of demyelination induced by ethidium bromide (EB). The chitosan nanoparticle dose was given alone after the induction of EB in rats that were categorized into demyelination and remyelination groups. According to the results, following the LINGO-1-directed siRNA–chitosan nanoparticle treatment, the animals performed better than controls. Specifically, the remyelination-treated group showed better motor performance than the demyelination group. LINGO-1 downregulation was associated with signs of repair in histopathological sections, higher expression of pontine myelin basic protein (MBP) messenger RNA (mRNA) and protein, and lower levels of caspase-3 activity, indicating neuroprotection and remyelination enhancement.

## 4. Conclusions

Drug delivery into the CNS is one of the most inhibitory points in the treatment of MS. Nanotechnology opened a new window for the treatment of MS and created promising opportunities by providing drug delivery nanosystems, as well as tolerance-inducing nanocarriers. On the one hand, several novel promising drug delivery systems are being developed for the targeted delivery of MS therapeutics and remyelinating agents into the CNS, in order to increase their therapeutic efficiency and decrease the unwanted side effects that are caused by the high doses. On the other hand, tolerance-inducing nanocarriers can be used as promising vaccines for antigen-specific immunotherapy. Currently, numerous studies describe the successful administration and the promising results of various nanoparticles, both lipidic and polymeric, in the EAE animal model at the preclinical stage, although there is a lack of clinical data in MS patients. Finally, the great potential in the improvement of nanotechnological formulations via the proper monitoring of their physical characteristics or the right choice and functionalization of their biomaterials continually creates future perspectives for the upgrade of current MS therapies.
